# Full spectrum flow cytometry-powered comprehensive analysis of PBMC as biomarkers for immunotherapy in NSCLC with EGFR-TKI resistance

**DOI:** 10.1186/s12575-023-00215-0

**Published:** 2023-07-24

**Authors:** Juan Zhou, Xiangling Chu, Jing Zhao, Mengqing Xie, Jing Wu, Xin Yu, Yujia Fang, Yazhou Li, Xiyan Li, Chunxia Su

**Affiliations:** 1 Department of Oncology, Department of Clinical Research Center, Shanghai Pulmonary Hospital &, Thoracic Cancer Institute, Tongji University School of Medicine, Shanghai, 200043 China; 2 Righton Biotechnology Co., Ltd, Shanghai, China

**Keywords:** Non-small cell lung cancer (NSCLC), Epidermal growth factor receptor-tyrosine kinase inhibitor (EGFR-TKI) resistance, Immune checkpoint inhibitor (ICI) plus chemotherapy, Peripheral blood mononuclear cell (PBMC)

## Abstract

**Background:**

Clinical studies suggest that immune checkpoint inhibitor (ICI) monotherapy has limited benefits in non-small cell lung cancer (NSCLC) patients after epidermal growth factor receptor-tyrosine kinase inhibitor (EGFR-TKI) failure. However, data about efficacy of ICI plus chemotherapy remain controversial, probably attributed to the heterogeneity among such population, and robust efficacy biomarkers are urgent to explore.

**Methods:**

A total of 60 eligible patients who received ICI plus chemotherapy after EGFR-TKI treatment failure were enrolled, 24 of whom peripheral blood mononuclear cell (PBMC) samples were collected at baseline and after 2 cycles of treatment. We have designed a 23-color-antibody panel to detect PBMC by full spectrum flow cytometry.

**Results:**

For EGFR-TKI resistant NSCLC patients: 1) ICI plus chemotherapy achieved an objective response rate (ORR) of 21.7% and a median progression-free survival (PFS) of 6.4 months. 2) clinical characteristics associated with worse efficacy included liver metastasis and platelet-to-lymphocyte ratio (PLR) > 200. 3) the proportion of immune cell subset associated with better efficacy was higher baseline effective CD4^+^T cells (E4). 4) the baseline expression of immune checkpoint proteins (ICPs) on cell subsets associated with better efficacy included: higher expression of CD25 on dendritic cells (DC) and central memory CD8^+^T cells (CM8), and higher expression of Lymphocyte activation gene 3 (LAG-3) on effective memory CD8^+^T cells (EM8). 5) the expression of ICPs after 2 cycles of treatment associated with better efficacy included: higher expression of CD25 on CD8^+^T/EM8 /natural killer (NK) cells. 6) the dynamic changes of ICPs expression associated with worse efficacy included: significantly decrease of T cell immunoglobulin and ITIM domain (TIGIT) expression on regular T cells (Tregs) and decrease of V-domain immunoglobulin suppressor of T cell activation (VISTA) expression on Th1. 7) a prediction model for the efficacy of ICI plus chemotherapy was successfully constructed with a sensitivity of 62.5%, specificity of 100%, and area under curve (AUC) = 0.817.

**Conclusions:**

Some EGFR-TKI-resistant NSCLC patients could indeed benefit from ICI plus chemotherapy, but most patients are primary resistant to immunotherapy. Comprehensive analysis of peripheral immune cells using full spectrum flow cytometry showed that compared to the proportion of cell subsets, the expression type and level of ICPs on immune cells, especially CD25, were significantly correlated with the efficacy of immunotherapy.

**Supplementary Information:**

The online version contains supplementary material available at 10.1186/s12575-023-00215-0.

## Introduction

Epidermal growth factor receptor (EGFR) gene mutation is the predominant molecular subtype of Asian non-small cell lung cancer (NSCLC) population, occurring in about 50% of patients [1]. The iterative development of EGFR-tyrosine kinase inhibitor (EGFR-TKI) has significantly extended the survival time of advanced NSCLC patients with EGFR mutation. The median overall survival (OS) of such patients could reach to around 3 years under the first-line third-generation EGFR-TKI treatment or sequencing treatment of first-/second-generation EGFR-TKI to third-generation EGFR-TKI [2]. However, further survival benefits are limited by the inevitable resistance to EGFR-TKI, the mechanism of which can be divided to EGFR-dependent and EGFR-independent ways. Resistance mechanisms conferring by secondary EGFR mutation have been widely studied [3]. For resistance to first- and second-generation EGFR-TKI, the most common mutation is T790M, accounting for approximately 50–60% of cases, which can be targeted by third-generation EGFR-TKI [4]. C797S is the most acquired mutation mediating resistance to third-generation EGFR-TKI, with 15%-40% of incidence, and fourth-generation EGFR-TKI aimed to target C797S is under phase I/II clinical trial [2]. Other acquired EGFR-mutations including L792X, G769X, G796R/D/S, V834L, M766Q, 20 insertion, L718Q, and G724S mutations are also reported to occur in 10%-20% cases after osimertinib resistance, but no targeted drugs are developed so far [3, 5]. Some EGFR-independent mechanisms are also clarified, such as MET/HER2/FGFR/PI3KCA/BRAF/KRAS amplification or mutation, RET/ALK/FGFR/NTRK fusion, cell cycle gene alterations, and histological transformation, and corresponding overcoming strategies are under investigation [5, 6]. However, the resistance mechanisms in 30%-50% of patients are still not clear now, creating a huge obstacle on the path to longer survival for these population and leaving a significant unmet need for clinical treatment.

In the last decade, the treatment pattern based on immune checkpoint inhibitor (ICI) targeting programmed cell death ligand 1 (PD-1) and programmed cell death ligand 1 (PD-L1), has achieved significant clinical efficacy in advanced and locally advanced NSCLC patients with driver gene negative and become the standard treatment for such population [7]. However, for patients with EGFR mutation, the study results showed different situation. A systematic retrospective analysis of five clinical studies, including OAK, POPLAR, CheckMate-017, CheckMate-057 and KEYNOTE-010, indicated that compared with docetaxel, survival benefits from second-line ICI monotherapy were mainly observed in EGFR wild-type patients, while not in patients with EGFR mutations (hazard ratio (HR), 1.11, 95% CI, 0.80–1.53, *P* = 0.54) [8]. A famous prospective phase II study (NCT02879994) which aimed to explore the efficacy of pembrolizumab in naïve patients with EGFR mutation was ceased after enrolling 11 patients, because only one patient responded whose confirmatory tests on EGFR gene even suggested wild type [9]. Whereas, the ATLANTIC study [10] found that duvalumab was moderately effective in EGFR-mutated NSCLC patients who had received at least two kinds of systemic therapies including EGFR-TKI, especially in patients with PD-L1 tumor cell score (TC) ≥ 25% (TC ≥ 25% vs. TC < 25%, median OS: 13.3 vs. 9.9 months), suggesting that pre-treatment of other therapies may have impact on the efficacy of immunotherapy. As indicated by retrospective subgroup analysis from IMpower 150 study [11], NSCLC patients with EGFR mutation and TKI resistance derived progression-free survival (PFS) (9.7 vs. 6.1 months, HR,0.59) and OS (29.4 vs. 18.1 months, HR,0.6) benefits from combination therapy of atezolizumab, bevacizumab, and chemotherapy, compared with bevacizumab plus chemotherapy. The results of phase III study ORIENT-31 [12] prospectively validated that the combination therapy of ICI, anti-angiogenesis, and chemotherapy could bring significant survival benefits to NSCLC patients with EGFR-TKI resistance (median PFS: 7.2 vs. 4.3 months, HR,0.59), compared to standard chemotherapy. The data of ORIENT-31 study also showed that ICI plus chemotherapy could also prolong median PFS of EGFR-TKI resistant patients. Moreover, a phase II study suggested that toripalimab plus chemotherapy was effective in NSCLC patients with EGFR mutation who did not acquire T790M mutation after EGFR-TKI failure (median PFS: 7.0 months, ORR:50%) [13]. Another phase II study (NCT04405674) proved the promising effect of tislelizumab plus chemotherapy in EGFR-TKI resistant patients with an ORR of 56.5% and a 1-year OS rate of 74.5%. Pembrolizumab plus chemotherapy was also demonstrated effective in such patients, which achieved an ORR of 42.3% and a median PFS of 8.3 months (NCT03242915). However, the results of CheckMate-722 (NCT02864251), a phase III study, showed that nivolumab plus chemotherapy failed to improve PFS (5.6 vs. 5.4 month, HR,0.75) and OS (19.4 vs. 15.9 month, HR,0.82) of patients with EGFR-TKI resistance, compared with chemotherapy. Another phase III study KEYNOTE-789 [14] (NCT03515837), which aimed to compare the efficacy of pembrolizumab plus chemotherapy and chemotherapy in patients with EGFR-TKI resistance, was also claimed failure. Thus, the efficacy of ICI plus chemotherapy in NSCLC patients with EGFR-TKI resistance remains controversy, suggesting that this population may has large heterogeneity, and the searching for efficacy biomarkers might be an effective way to screen and enrich population to benefit and improve the efficiency of treatment.

Compared to tissue biopsy, peripheral blood has become an important alternative sample source to explore biomarkers due to its non-invasive and convenient sampling [15]. Immune cells in peripheral blood, which served a critical role in systematic immune responses, were believed to have potential value in predicting the immunotherapy efficacy. In fact, more and more researches supported that the localized antitumor immune response cannot exist without continuous communication with the periphery and intact peripheral immune function, communication and trafficking are required for ICI efficacy [16]. Profiling of T cell clonotypes by single-cell sequencing of RNA and T cell receptors (TCR) in patients with different types of cancer revealed that clonotypic expansion of effector-like T cells were simultaneously detected in tumor, normal adjacent tissue, and peripheral blood. Effective immunotherapies could drive de novo peripheral immune responses culminating in new effector T cell infiltration to replenish the dysfunctional T cells in local tumor microenvironment (TME) [17]. A study of neoadjuvant/adjuvant anti-PD-1 therapy in stage III/IV melanoma also demonstrated that T cell proliferation after ICI treatment could be early observed in peripheral blood and then recruited to tumor [18]. These studies consistently highlighted that the T cell response to ICI may originate outside the tumor and rely on peripheral T cell recruitment, which provided theoretical support to investigate the association between immune cells in peripheral blood and response to immunotherapy.

As so far, many kinds of immune cells in peripheral were found to be promising to predict immunotherapy efficacy in advanced NSCLC without driver gene mutation, such as the ratio of neutrophil-to-lymphocyte (NLR), TCR clonality and diversity of T cells, memory T cells, and the amount and function of CD8^+^T cells or immunosuppressive cells eg. regular T cells (Tregs) and myeloid-derived suppressive cells (MDSCs) [19]. However, in patients with EGFR-TKI resistant NSCLC, there is still a lack of relevant explorations on the association between peripheral blood immune cells and immunotherapy efficacy. Therefore, this study aimed to search potential efficacy biomarkers for EGFR-TKI resistant NSCLC patients who received ICI plus chemotherapy from the perspective of immune cells in peripheral blood.

## Methods

### Patient enrollment

Patients who have advanced NSCLC with metastatic/recurrent or unresectable stages were enrolled into this study from Shanghai Pulmonary Hospital between June 2018, and June 2022. Eligible patients were as follows: 1) confirmed NSCLC by pathology; 2) staged IV or unresectable IIIB-IIIC according to the eighth edition of the TNM classification for lung cancer; 3) EGFR sensitive mutation, including 19DEL, L858R, G719X, L861Q and S768I, was confirmed by amplification refractory mutation system-polymerase chain reaction (ARMS-PCR) or next-generation sequencing (NGS); 4) failed to EGFR-TKI treatment, including patients with disease progression and confirmed T790M-negative after first-/second-generation of EGFR-TKI, or T790M-positive but progressed further after third-generation of EGFR-TKI, and patients who progressed after third-generation of EGFR-TKI as first-line therapy; 5) measurable lesions according to Response Evaluation Criteria in Solid Tumors version 1.1 (RECIST v1.1); 6) Eastern Cooperative Oncology Group (ECOG) performance status 0–2; 7) expected survival ≥ 3 months; and 8) received PD-1 inhibitor plus chemotherapy and efficacy data was available. Exclusion criteria were followed: 1) with known co-mutations; 2) discontinued EGFR-TKI therapy due to intolerable side effects, or other factors not related to disease progression; 3) active multiple primary malignancies diagnosed within 5 years prior to treatment; 4) autoimmune diseases requiring systemic treatment within 2 years; 5) received other immunotherapy including but not limiting vaccines and adoptive cellular immunotherapy; and 6) receiving intensive immunosuppressive agents. Efficacy was evaluated according to RECIST v1.1. Objective response rate (ORR) was defined as the proportion of patients who had complete response and partial response (PR) to treatment. Disease control rate (DCR) was defined as the proportion of patients who had complete response, PR, or stable disease (SD) to treatment. PFS was defined as the interval from the initiation of ICI plus chemotherapy to confirmed disease progression or death of any cause. Similar to the concept of durable clinical benefits promoted by previous study [20], we defined clinical benefits (CB) from ICI plus chemotherapy in patients with EGFR-TKI resistant NSCLC as PFS of at least 6 months and non-benefit (NB) from combination treatment as PFS < 6 months, with reference of median PFS in our cohort.

### Sample collection and detection

We have collected 10 ml whole blood samples from 24 eligible patients at baseline and after 2 cycles of ICI plus chemotherapy using sterile anticoagulant tubes. PBMC was extracted by density gradient centrifugation and stored at -80 C.

The immunophenotyping of major cell subsets presented in PBMC samples were tested by full spectrum flow cytometry (Cytek NL-CLC), which was developed to better achieve a multiparameter analysis by measuring the emitted fluorescence for all probes across the full-spectrum from each cell with 3 lasers and 38 channels sequential 3 avalanche photodiodes (APD) units after dispersion with prism that have higher photoelectric conversion efficiency in a wider range of light waves compared to photomultiplier tube (PMT), and extract the signals based on the spectral shape of each fluoroprobe using unique algorithm in high speed, high sensitive, accurate, automatic and real-time. While this novel technology has been in use in research settings for several years, it is just beginning to emerge in clinical markets. To the best of our knowledge, it has only been approved to clinical use in hematological system diseases diagnosis up to date. In this study, we have designed a 23-color-antibody panel to profile the immune cells in peripheral blood (Supplement Table 1). In addition, we used % parent and mean fluorescence intensity (MFI) for separately quantitative evaluation of immune cell subsets and immune checkpoint proteins (ICPs), and detailed gating strategy for detection data from full spectrum flow cytometry was shown in supplement Fig. 1.

**Table 1:** Baseline characteristics of all patients enrolled to this study

Characteristics	Total (*N* = 60)	CB (*N* = 21)	NB (*N* = 39)	*P-value*
**Age(year)**				0.108
median(range)	63.5 (19–76)	59 (19–76)	65 (39–76)	
**Sex**				0.935
male	31 (51.7)	11 (52.4)	20 (51.3)	
female	29 (48.3)	10 (47.6)	19 (48.7)	
**Smoking**				0.473
never	51 (85.0)	19 (90.5)	32 (82.1)	
ever	9 (15.0)	2 (9.5)	7 (18.4)	
**Histology**				0.119
Adeno	56 (93.3)	18 (85.7)	38 (97.4)	
NOS	4 (6.7)	3 (14.3)	1 (2.6)	
**Number of distant metastases**				0.217
0–1	35 (58.3)	15 (71.4)	20 (51.3)	
2–3	21 (35.0)	6 (28.6)	15 (39.5)	
> 3	4 (6.7)	0 (0.0)	4 (10.5)	
**Organ metastasis**				
bone	24 (40.0)	8 (38.1)	16 (41.0)	0.825
brain	13 (21.7)	4 (19.0)	9 (23.1)	1.000
liver	4 (6.7)	0 (0.0)	4 (10.3)	0.287
**EGFR mutation**				0.609
19DEL	30 (50.0)	10 (47.6)	20 (51.3)	
L858R	27 (45.0)	9 (42.9)	18 (46.2)	
G719X	3 (5.0)	2 (9.5)	1 (2.6)	
**Acquired T790M**				0.470
no	50 (83.3)	19 (90.5)	31 (79.5)	
yes	10 (16.7)	2 (9.5)	8 (20.5)	
**Other treatment**^**a**^				0.129
no	38 (63.3)	16 (76.2)	22 (56.4)	
yes	22 (36.7)	5 (23.8)	17 (43.6)	
**PD-L1**				0.914
not clear	52 (86.7)	19 (90.5)	33 (84.6)	
negative	4 (6.7)	1 (4.8)	3 (7.7)	
positive	4 (6.7)	1 (4.8)	3 (7.7)	
**ICI line**				0.185
2–3	48 (80.0)	19 (90.5)	29 (74.4)	
> = 4	12 (20.0)	2 (9.5)	10 (25.6)	
**ICI drug**				0.193
pembrolizumab	12 (20.0)	4 (19.0)	8 (20.5)	
nivolumab	3 (5.0)	1 (4.8)	2 (5.1)	
camrelizumab	7 (11.7)	3 (14.3)	4 (10.3)	
toripalimab	9 (15.0)	3 (14.3)	6 (15.9)	
tislelizumab	6 (10.0)	2 (9.5)	4 (10.3)	
sintilimab	8 (13.3)	2 (9.5)	6 (15.9)	
not clear	15 (25.0)	6 (28.6)	9 (23.1)	

*Abbreviation*: *NOS* Not otherwise specified, *ICI* Immune checkpoint inhibitor

^a^Other treatments refer to patients who had received other systemic treatments, including but not limited to chemotherapy, antiangiogenic therapy, etc. after the failure of EGFR-TKI treatment and before ICI plus chemotherapy

**Fig. 1:**
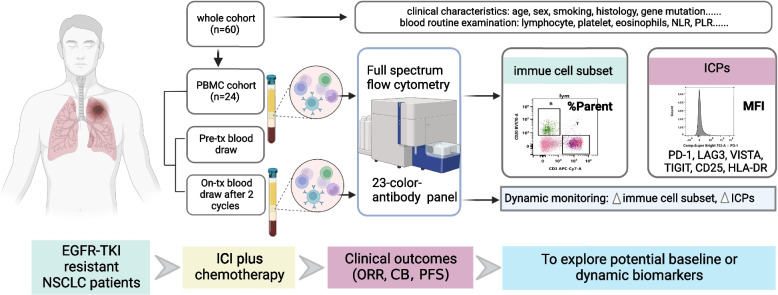
Flowchart of patient enrollment and study design. EGFR-TKI resistant NSCLC patients who received ICI plus chemotherapy were enrolled. The whole cohort include 60 patients and information of clinical characteristics and outcomes were collected. Among them, 24 patients were considered as PBMC cohort whose PBMC samples at baseline and 2 cycles of ICI plus chemotherapy were collected. All PBMC samples were detected through full spectrum flow cytometry and a 23-color-antibody panel was designed to profile the immune cells in peripheral blood. Abbreviations: NLR, neutrophil to lymphocyte ratio. PLR, platelet-to-lymphocyte ratio. Pre-tx, pre-treatment. On-tx, on treatment. PBMC, peripheral blood mononuclear cell. ICPs, immune checkpoint proteins. MFI, mean fluorescence intensity. EGFR-TKI, epidermal growth factor receptor-tyrosine kinase inhibitor. NSCLC, non-small cell lung cancer. ICI, immune checkpoint inhibitor. ORR, objective response rate. CB, clinical benefits. PFS, progression-free survival

### Statistical analysis

SPSS 22.0 version and R 4.2.2 were used for statistical analysis. Median and quartile ranges were used to characterize quantitative variables. Mann–Whitney U and t-test were respectively used to examine differences between subgroups of quantitative variables with non-normal distribution and normal distribution. Chi-square test or Fisher's exact test was used to examine differences between subgroups of categorical variables. Due to the small sample size and many indexes in this study, *P* < 0.1 was used for preliminary variable screening, and to avoid high false positivity, multiple hypothesis test correction was performed and q-value < 0.1 was used for final differential variable confirmation. The Kaplan–Meier method was used to estimate PFS, the Log-rank method was used to test the differences between groups, and the Cox proportional hazard model was used to analyze the univariate/multivariate effects. X-title was used to calculate the cut-off value of continuous variables associated with the survival. Lasso regression was used to screen factors related to the treatment response and Logistic regression was used to construct the prediction model. *P* < 0.05 on both sides was considered statistically significant.

## Results

### Patient characteristics

A total of 60 advanced NSCLC patients with EGFR-TKI resistance were enrolled in the study (Fig. 1). The baseline characteristics of all participants were summarized in Table 1. The median age was 63.5 years old, and Most patients had no history of smoking (85%). All patients were diagnosed with lung adenocarcinoma except four patients with carcinoma not otherwise specified (NOS). The most common subtypes of EGFR mutations were 19DEL and L858R, and three patients with G719X were included. The higher proportions of patients with bone metastases and brain metastases were probably attributed to the fact that all patients had underwent at least one kind of systematic treatment. It was worth noting that only 16.7% of patients in this study had acquired T790M, probably caused by preference selection of clinicians as some research data suggested that negative T790M may be a favorable factor for immunotherapy in NSCLC patients who failed EGFR-TKI treatment [21]. There were 80% of patients who received ICI plus chemotherapy at second or third line, and only 36.7% of patients had received other systematic treatment before immunotherapy. As PD-L1 is not required to be tested for such patients before immunotherapy, the expression of PD-L1 was not evaluated in 86.7% of patients in this study, so we did not make subsequent analysis on PD-L1 expression.

### Efficacy and influencing clinical factors of ICI plus chemotherapy

The median follow-up time was 19.7 months, and the median PFS of total population was 6.4 months (95%CI: 4.3–8.6), the overall ORR and DCR were 21.7%, and 86.7%, respectively, and the CB rate as we previously defined was 31.6%.

**Fig. 2:**
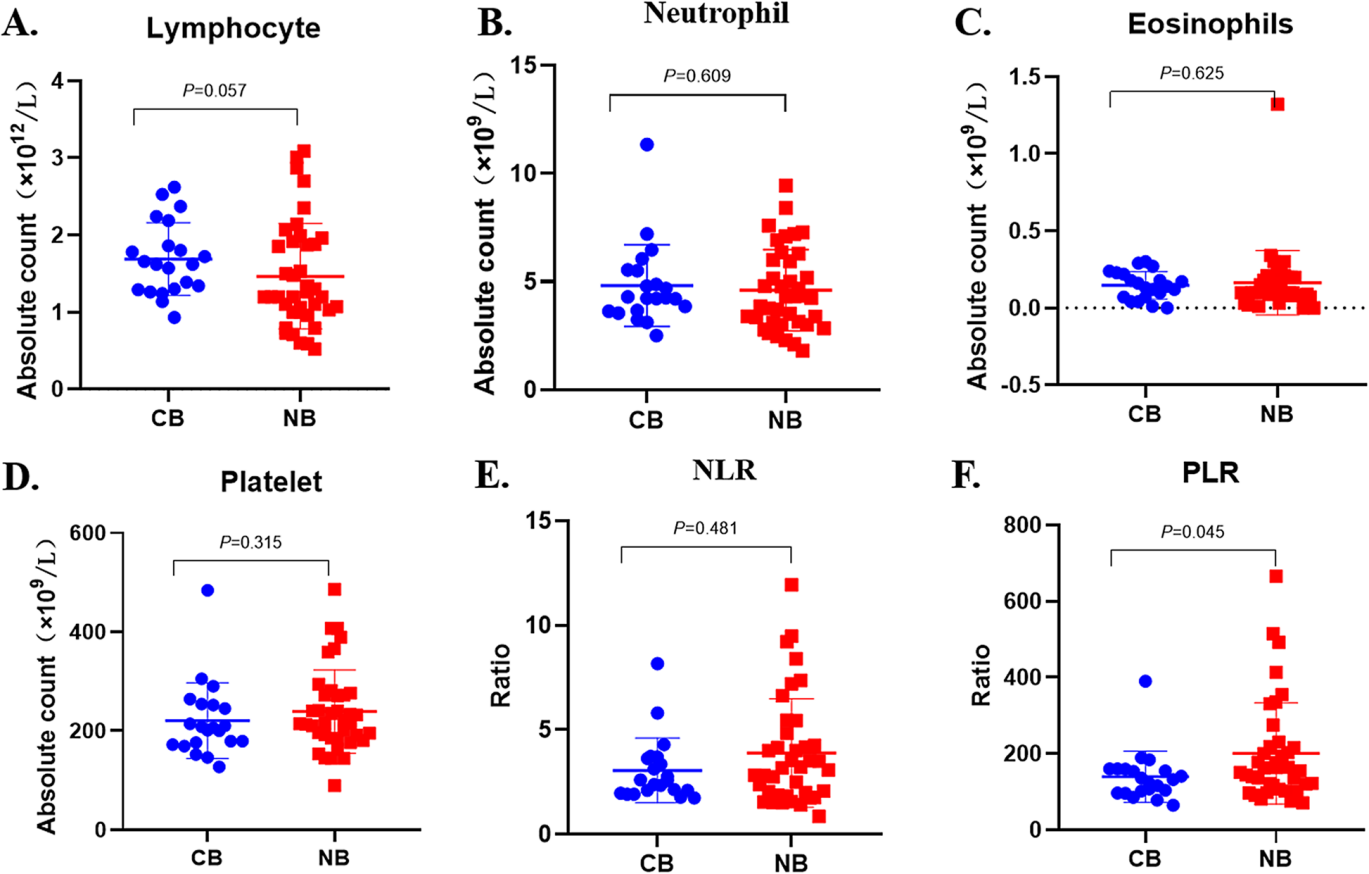
The association of baseline hematologic indicators with efficacy of ICI plus chemotherapy. Comparisons of baseline hematologic indicators between patients with clinical benefits (CB) and patients with non-benefit (NB) from ICI plus chemotherapy, including absolute lymphocyte count (**A**), absolute neutrophil count (**B**), absolute eosinophil count (**C**), platelet count (**D**), the ratio of neutrophil-to-lymphocyte (NLR) (E), and the ratio of platelet -to-lymphocyte (PLR)

To explore the potential clinical factors associated with outcome of ICI plus chemotherapy, we firstly compared the baseline characteristics between CB and NB groups, and found no difference in all common characteristics including age, sex, smoking history, pathology, distant metastasis, gene mutation subtype, T790M mutation, PD-L1 expression, and immunotherapy treatment line. Previous studies reported that baseline NLR, eosinophils, platelets, and platelet-to-lymphocyte ratio (PLR) were associated with immunotherapy efficacy in advanced NSCLC patients [22, 23]. Therefore, we further compared these baseline hematologic indicators between the two groups, and found no significant difference in the absolute count of lymphocytes (Fig. 2A), neutrophils (Fig. 2B), eosinophils (Fig. 2C), platelets (Fig. 2D), and the NLR (Fig. 2E), while the PLR in CB group was significantly lower than that in NB group (*P* = 0.045) (Fig. 2F).

To analyze the impact of such clinical factors on PFS of ICI plus chemotherapy, we firstly converted the above continuous variables, such as the absolute counts of lymphocytes, neutrophils, eosinophils, platelets, NLR, and PLR, to categorical variables according to the optimal cut-off values of 1.07, 3.5, 215, 4, and 200, respectively, calculated by X-Title. Univariate analysis of PFS in the total population showed that some variables, including the number of distant metastatic organs, liver metastasis, other treatments before immunotherapy, the lines of immunotherapy, lymphocytes, NLR, and PLR, were significantly related with PFS (Table 2). All variables with *P* < 0.1 in univariate analysis was included in the multivariate analysis and taking collinearity between any two of these variables into account meanwhile, we used Cox regression with forward stepwise (likelihood ratio) to perform multivariate analysis. The results suggested that only “liver metastasis” (*P* < 0.001) and “PLR” (*P* = 0.003) were independently associated with PFS of EGFR-TKI resistant NSCLC patients receiving ICI plus chemotherapy.

**Table 2:** Univariate and multivariate analysis of PFS of all patients receiving ICI plus chemotherapy

Characteristics	Univariate			Multivariate*		
	**HR**	**95%CI**	***P-value***	**HR**	**95%CI**	***P-value***
**Age(year)** (< = 63.5/ > 63.5)	1.119	0.573–2.186	0.741			
**Sex** (male/female)	1.068	0.558–2.043	0.843			
**Smoking**(never/ever)	1.518	0.462–4.987	0.491			
**Histology(**adeno/NOS)	0.955	0.333–2.741	0.932			
**Number of distant metastases**						
0–1/ > 3	0.097	0.024–0.397	0.001			
2–3/ > 3	0.171	0.042–0.691	0.013			
**Bone metastasis**(no/yes)	0.905	0.468–1.748	0.766			
**Brain metastasis**(no/yes)	0.611	0.273–1.368	0.231			
**Liver metastasis**(no/yes)	0.096	0.028–0.333	< 0.001	0.060	0.016–0.226	< 0.001
**EGFR mutation**						
L858R/19DEL	0.736	0.376–1.441	0.372			
G719X/19DEL	0.964	0.284–3.279	0.953			
**Acquired T790M**(no/yes)	0.614	0.268–1.410	0.250			
**Other treatment**(no/yes)	0.426	0.209–0.868	0.019			
**ICI line**(2–3/ > = 4)	0.273	0.123–0.603	0.001			
**Lymphocyte**(< = 1.07/ > 1.07)	2.745	1.312–5.746	0.007			
**Neutrophile**(< = 3.5/ > 3.5)	1.263	0.621–2.570	0.519			
**Eosinophils**(< = 0.16/ > 0.16)	1.454	0.732–2.887	0.286			
**Platelet**(< = 215/ > 215)	0.647	0.340–1.229	0.183			
**PLR(< = 200/ > 200)**	0.335	0.133–0.842	0.020	0.229	0.085–0.616	0.003
**NLR(< = 4/ > 4)**	0.492	0.239–1.014	0.055			

*Abbreviation*: *NOS* Not otherwise specified, *ICI* Immune checkpoint inhibitor, *PLR* Platelet-to-lymphocyte ratio, *NLR* Neutrophil-to-lymphocyte

*All variables with *P* < 0.1 in univariate analysis was included in the multivariate analysis. Cox regression with forward stepwise (likelihood ratio) was used to perform multivariate analysis

### Profiling of immune cells in peripheral blood

#### Characteristics of patients collected PBMC

Among all 60 patients in this study, PBMC samples were collected from 24 patients at the baseline and after 2 cycles of ICI plus chemotherapy. The median interval between the two sampling was 50 days with range of 25–69 days.

In order to find out whether there was large population selection bias in these 24 patients with collected specimens, we summarized their baseline characteristics and found that almost all clinical characteristics were consistent with those of the whole study population (Supplement Table 2), suggesting that there was no significant bias in this population. Moreover, all these 24 patients had no smoking history, and 9 of them were divided into the CB group, while 15 of them were divided into the NB group according to our definition before. The baseline characteristics in the two groups were balanced. The median PFS of all these 24 patients was 5.32 months (95%CI, 3.97–6.68). Both univariate and multivariate analyses suggested that liver metastasis was the independent factor for poor prognosis in patients with EGFR-TKI resistant NSCLC (*P* = 0.033) (Supplement table 3).

#### Proportion of immune cell subsets

**Fig. 3:**
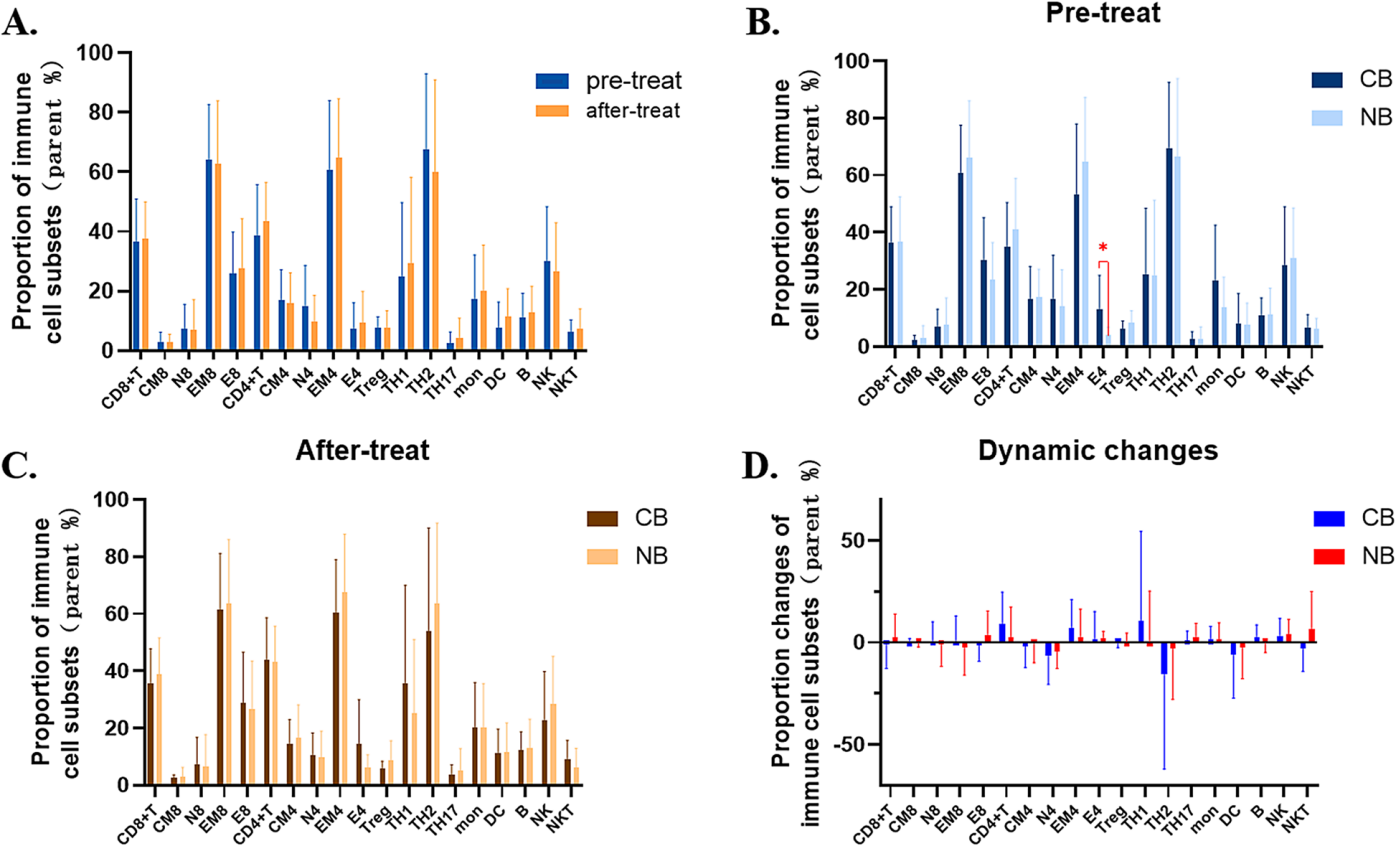
Association of immune cell subsets with efficacy of ICI plus chemotherapy. Comparisons of immune cell subsets proportions in different subgroups: Indicators comparison between patients pre- and after-treatment (**A**). Pre-treat indicators comparison between patients in CB and NB groups (**B**). On-treatment indicators comparison between patients in CB and NB groups (**C**). Dynamic changes of indicators comparison between patients in CB and NB groups (**D**). Abbreviations: CB, clinical benefits, NB, non-benefit. *P < 0.1

A total of 19 antibodies were designed to cluster common peripheral blood immune cells according to their development lineage in this study and only 19 immune cell subsets were finally identified after screening out subsets that could not be detected in more than 10% of the patients. Firstly, we compared the proportions of immune cell subsets in patients before and after receiving ICI plus chemotherapy and found that there was no significant change after 2 cycles of treatment (Fig. 3A). Then, we respectively compared the difference in the proportions of immune cell subsets in the CB and NB groups, and found that: at baseline, compared with NB group, the CB group had a significantly higher proportion of effective CD4^+^T cell (E4, CD4^+^CD45RA^+^CD197^−^), with marginal statistical significance (*P* = 0.055) (Fig. 3B), while after 2 cycles of treatment, there was no significant difference in the proportions of immune cell subsets (Fig. 3C). In addition, there was also no significant difference in the dynamic changes of immune cell subsets proportions between the CB and NB groups (Fig. 3D).

#### Expression of immune checkpoint proteins

Many studies suggested that PD-1 expression on some subsets of peripheral immune cells, like CD8^+^ T cell [24], CD4^+^ T cell [25], NK cells [26], etc. were associated with immunotherapy outcomes. Thus, we added PD-1 antibody into our panel. Besides, as novel immune checkpoint molecules on T cells have been discovered continuously and previous studies have suggested their alternative roles for immune escape to PD-1/PD-L1 pathway [27], we would like to supplement some antibodies against these novel ICPs into our multi-color panel. Initially, some of these checkpoint targets under clinical trials [28] were selected, such as lymphocyte activation gene 3 (LAG-3), T cell immunoglobulin and mucin domain-containing protein 3 (TIM-3), V-domain immunoglobulin suppressor of T cell activation (VISTA), and T cell immunoglobulin and ITIM domain (TIGIT). However, TIM-3 was given up due to the unsatisfying staining in preliminary experiment. In addition, we noticed that CD25 [29] and human leukocyte antigen DR (HLA-DR) [30] in our designed panel could not only be used as clustering marker for Tregs and dendritic cells (DC) respectively, but also be regarded as ICPs on immune cell subsets, so we have also evaluated their expressions on immune cell subsets as ICPs using MFI. The overall expressions of these ICPs are shown in supplement Fig. 2. As can be intuitively seen from the figure, the ICPs expressions on immune cell subsets seem higher in the CB group than those in NB group, no matter at baseline or after 2 cycles of treatment.

**Fig. 4:**
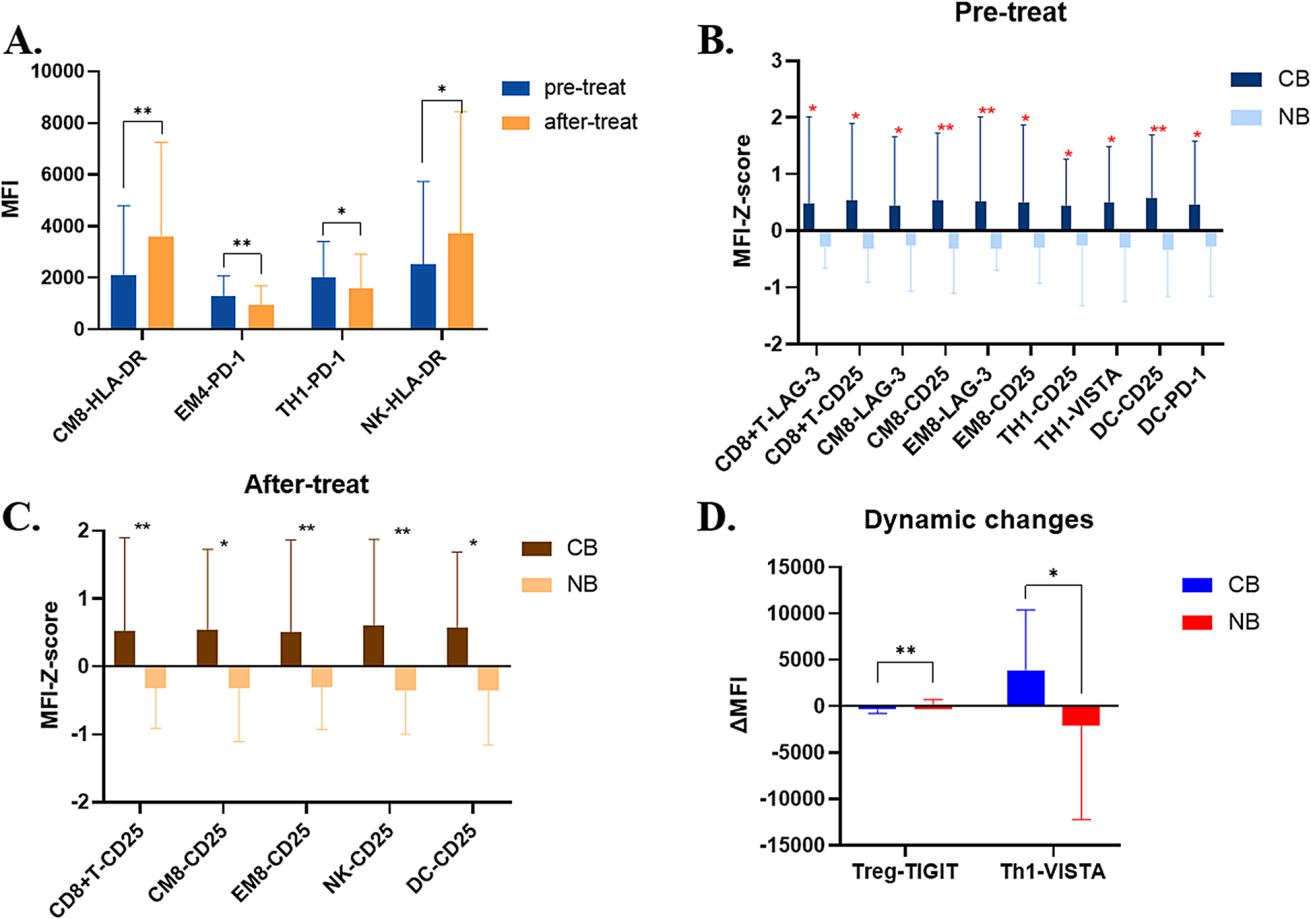
Association of immune checkpoint proteins with efficacy of ICI plus chemotherapy. Comparisons of immune checkpoint proteins (ICPs) expressions on immune cell subsets in different subgroups: Indicators comparison between patients pre- and after-treatment (**A**). Pre-treat indicators comparison between patients in CB and NB groups (**B**). After-treat indicators comparison between patients in CB and NB groups (**C**). Dynamic changes of indicators comparison between patients in CB and NB groups (**D**). Abbreviations: CB, clinical benefits, NB, non-benefit. *P < 0.1, ** *P* < 0.05

To find the specific differential variables, we made further statistical analysis. Firstly, we compared the expression levels of ICPs on immune cell subsets before and after receiving ICI plus chemotherapy in all patients, and the data showed that after treatment, HLA-DR on central memory CD8^+^T cell (CM8, CD8^+^ CD45RA^−^CD197 ^+^) were significantly elevated, while PD-1 on effective memory CD4^+^T cell (EM4, CD4^+^ CD45RA^−^CD197 ^−^) was significantly decreased (Fig. 4A). Secondly, we made further comparison between CB group and NB group, and found that: at baseline, CD25 expression on CM8 and DC, and LAG-3 expression on effective memory CD8^+^T cell (EM8, CD8 + CD45RA^−^CD197^−^) were significantly higher in CB group than in NB group (Fig. 4B), while after treatment, CD25 expression on CD8^+^T/EM8/natural killer (NK) cells were significantly higher in CB group than in NB group (Fig. 4C). Lastly, the dynamic changes comparison suggested that the decrease of TIGIT on Tregs was more significant in NB group than in CB group, and the VISTA on Th1 was increased in CB group but decreased in NB group (Fig. 4D).

### Construction of model to predict efficacy of ICI plus chemotherapy

The above data suggested that some clinical characteristics and features of peripheral blood immune cells were associated with the efficacy of EGFR-TKI resistant NSCLC patients receiving ICI plus chemotherapy. Therefore, Lasso regression was used to systematically analyze the potential influencing factors, and all differential variables between CB and NB group with *P* < 0.1 were included. The results showed that when the parameter λ was the minimum value, variables including platelet, pre-treatment E4, and on-treatment CD25 on NK cells were confirmed to be meaningful to predict CB response.

**Fig. 5:**
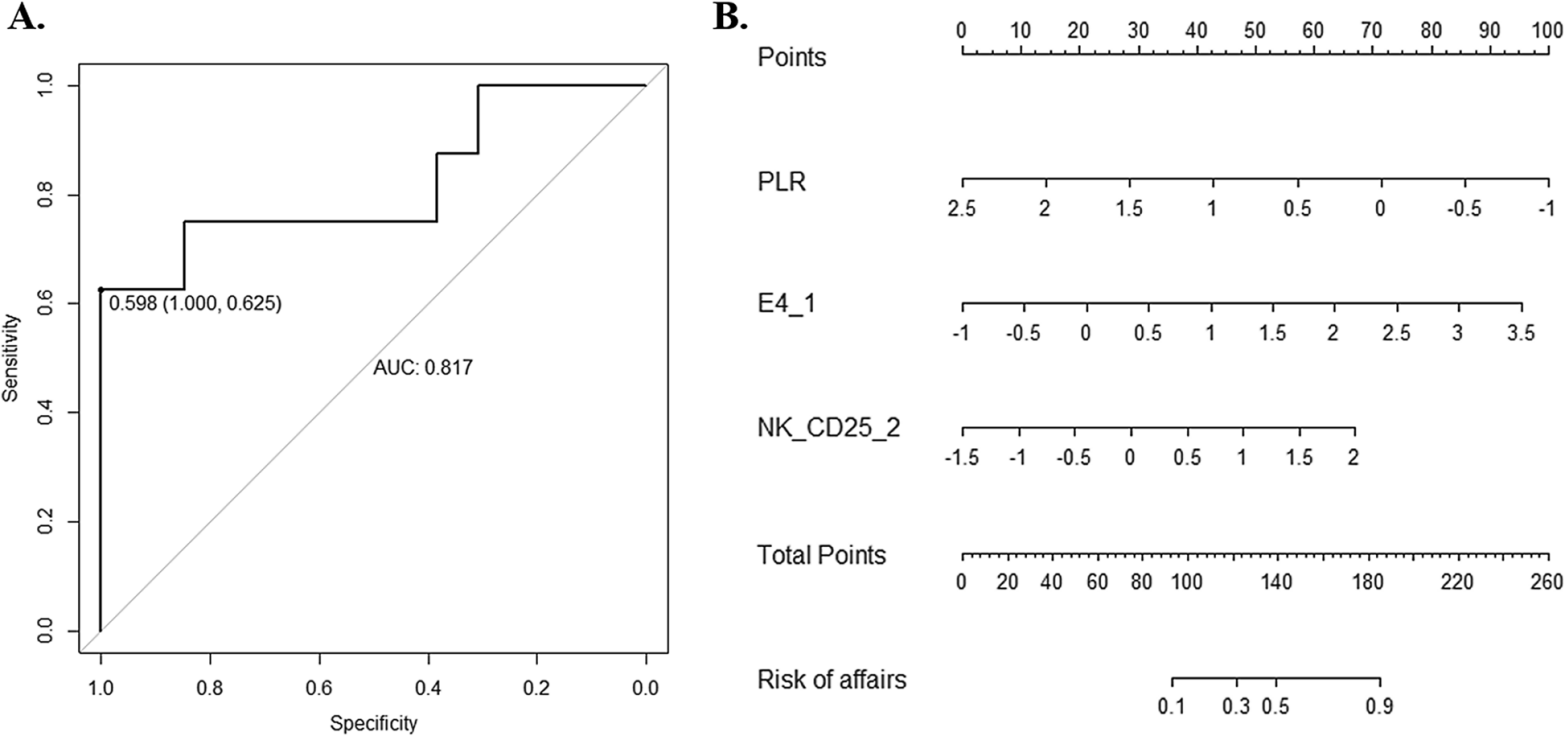
Prediction model to efficacy of ICI plus chemotherapy. **A** is the receiver operating characteristic (ROC) curve of the prediction model, with an AUC area of 0.817. When the cut-off value of model score is 0.598, the sensitivity is 62.5% and the specificity is 100%. **B** is the Nomo diagram to display the prediction model. Factors contributed to the prediction model include PLR, pre-treatment E4 (E4_1), and after-treatment CD25 on NK cells (NK_25_2). Lower value of PLR and higher values of E4_1 and NK_25_2 bring higher points, and higher points predict higher probability to acquire clinical benefit (CB) from ICI plus chemotherapy for patients with EGFR-TKI resistance

Then, Logistic regression was used to establish a prediction model. Internal cross-validation data suggested that when the model score threshold was 0.598, the sensitivity and specificity of the model were 62.5% and 100%, respectively, with area under curve (AUC) = 0.817 (Fig. 5A). A Nomo diagram was drawn to display this model, and the values of relevant quantitative variables were standardized by z-score transformation (Fig. 5B).

## Discussion

In this study, we investigated the efficacy of ICI plus chemotherapy for advanced NSCLC patients with EGFR-TKI resistance in a real-word setting and explored potential peripheral blood immune cells-related biomarkers through full spectrum flow cytometry. Our data showed that for EGFR-TKI resistant NSCLC patients: 1) ICI plus chemotherapy achieved an ORR of 21.7% and a median PFS of 6.4 months. 2) clinical characteristics associated with worse efficacy included liver metastasis and PLR > 200. 3) the proportion of immune cell subset associated with better efficacy was higher proportion of baseline E4. 4) the baseline expressions of ICPs on cell subsets associated with better efficacy included: higher expression of CD25 on DC and CM8, and higher expression of LAG3 on EM8. 5) the expressions of ICPs after 2 cycles of treatment associated with better efficacy included: higher expression of CD25 on CD8^+^T/EM8/NK cells. 6) the dynamic changes of ICPs expression associated with worse efficacy included: significantly decrease of TIGIT expression on Tregs and decrease of VISTA expression on Th1. 7) based on clinical characteristics and features of peripheral immune cells, a prediction model for the efficacy of ICI plus chemotherapy was successfully constructed with a sensitivity of 62.5%, specificity of 100%, and AUC = 0.817.

In recent years, more and more real-world studies have found that some EGFR-TKI-resistant NSCLC patients could indeed benefit from PD-1/PD-L1 antibody combined with chemotherapy, although anti-PD-1/PD-L1 monotherapy is demonstrated to be poor effective. For example, Hu et al. [31] found in 99 NSCLC patients with EGFR-TKI resistance that immunochemotherapy was significantly more effective than ICI monotherapy (median PFS: 5.0 vs. 3.0 months, *P* = 0.02; median OS: 19.0 vs. 7.4 months, *P* = 0.009). Sun et al. [32] (median PFS: 5.9 vs. 2.4 months, *P* = 0.001) and Tian et al. [33] both reported similar results (median PFS: 5.5 vs. 2.2 months,* P* = 0.002; median OS: 14.4 vs. 7.0 months, *P* = 0.001). Intriguingly, Tian et al. [33] also found that among EGFR-TKI-resistant NSCLC patients, those who received immunochemotherapy following TKI failure had a better survival than those who had also received other treatments before immunochemotherapy (median PFS: 7.2 vs. 3.4 months, *P* < 0.0001; median OS: 15.1 vs. 8.4 months, *P* < 0.0001). Our univariate analysis of the total population showed a similar trend, though multivariate analysis through Cox regression with forward stepwise (likelihood ratio) showed that this variable failed to step in the risk factor model, which might be attributed to the small sample size of the present study. But after literatures investigation about the optimal timing of immunotherapy [34, 35] and the potential impact of EGFR-TKI on tumor microenvironment [36, 37], we prone to believe that the earlier treatment of immunochemotherapy after EGFR-TKI resistance, the better therapeutic effect would be observed. Besides, Cheng et al. discovered that for EGFR-TKI-resistant NSCLC patients, immunochemotherapy was more effective in young patients without T790M, liver metastasis, and brain metastasis. Our study only suggested that immunochemotherapy was less effective in patients with liver metastasis, and we believe that this is not unique for EGFR-TKI-resistant NSCLC population as liver metastasis has been widely confirmed as an independent factor of treatment resistance and poor prognosis of lung cancer.

To identify more effective biomarkers to screen the real beneficiaries of EGFR-TKI-resistant NSCLC patients from ICI plus chemotherapy, we systematically analyzed the indicators in peripheral blood, which is now acknowledged as a vital important pool to explore biomarkers. Peripheral blood-related biomarkers to predict efficacy of immunotherapy has been widely studied in advanced NSCLC with drive gene negative, mainly including factors on the cellular level (eg. circulating tumor cells (CTC), immune cells, etc.), the DNA level (eg. circulating tumor DNA (ctDNA), gene mutations, etc.), and the protein level (eg. autoantibodies, cytokines, etc.). Among them, some indexes in blood routine examination, such as lymphocytes, neutrophils, eosinophils, platelets, derived NLR, PLR, etc. have attracted more attentions because of their easy acquisition. Diem et al. [22] found that high levels of NLR and PLR at baseline were associated with worse PFS and OS in patients with advanced NSCLC treated with nivolumab monotherapy. A meta-analysis that included 21 studies and 1845 patients also suggested that high baseline levels of NLR and PLR were associated with poor outcomes of immunotherapy. Likely, in our study, both univariate/multivariate analysis of PFS and lasso regression about treatment CB response suggested that higher baseline PLR (> = 200) was associated with worse outcome of ICI plus chemotherapy. There is accumulating evidence that platelets have an interaction with immune system and protect tumor cells from different cytotoxic lymphocytes including NK cells and effector T cells [38, 39]. A study from Hinterleitner, et al. [40] suggested that platelet deteriorated efficacy of ICI by loading PD-L1 transformed from tumor cell to inhibit CD4 and CD8 T-cells. Platelets can also support immunosuppressive TME by releasing cytokines like TGF-beta [41]. Therefore, the platelet inhibitors are believed to be promising in enhancing immunotherapy efficacy. But notably, a previous study reported that a decrease of platelet after 2 cycles of EGFR-TKI treatment correlated with the longer OS (HR = 0.293, 95%CI: 0.107–0.799, *P* = 0.017) [42]. A meta-analysis which enrolled a total of 2,889 patients in 12 studies receiving any treatments, suggested that patients with an elevated PLR were expected to have a shorter OS (HR = 1.492, 95% CI: 1.231–1.807, *P* < 0.001) [43]. Thus, we think that high platelet count is rather a prognostic factor than a predictive factor, as more studies reported that platelet can fuel tumor growth, invasion, and metastasis [44].

Full spectrum flow cytometry is a recently developed technology that captures the full emission spectrum of fluorescent molecules using arrays of highly sensitive light detectors, and allows for high-dimensional flow cytometric analyses of cells and particles in suspension. To date, full spectrum flow cytometry has enabled characterization of 50 parameters in a single sample. In this study, we firstly used full spectrum flow cytometry to comprehensive analyze the peripheral blood immune cells in EGFR-TKI-resistant NSCLC patients receiving ICI plus chemotherapy, and identified 19 subsets of immune cells and 6 kinds of ICPs expression on each subset. The results showed that many features of peripheral blood immune cells were related to the efficacy of ICI plus chemotherapy, and we further constructed an efficacy prediction model. The internal cross-validation of the model suggested that the sensitivity was 62.5%, the specificity was 100%, and the AUC was 0.817, but it still needed to be validated by an external cohort. Baseline E4 and on-treatment CD25 on NK cells were the two main factors contributed to our prediction model. The association between peripheral blood CD4^+^T cells and immunotherapy efficacy has been revealed in previous studies. Iwahori et al. [45] reported that the cytotoxicity of peripheral blood T cells was related to the proportion of EM4 and EM8 cells, while the cytotoxicity of peripheral blood T cells was related to the PFS of immunotherapy in patients with advanced NSCLC. Duchemann et al. [25] found that the ratio of CD8^+^PD-1^+^ T cells to CD4^+^PD-1^+^ T cells was associated with benefit from immunotherapy in the advanced NSCLC cohort. CD25, the alpha-chain of the heterotrimer IL-2 receptor, is expressed at high levels in many types of hematological malignancies but at low levels in most solid tumors [46]. CD25 is also highly expressed on activated circulating immune cells and Tregs, and as a potential target, new immunotherapeutic strategies including chimeric antigen receptor (CAR)-NK targeting CD25 [47] are currently being developed [48]. In our study, the high expression of CD25 on peripheral blood immune cells was detected in EGFR-TKI resistant NSCLC patients, suggesting that PD-1/PD-L1 monoclonal antibody combined with CD25 inhibitors may further improve the immunotherapy efficacy of this population.

To conclude, our study revealed that some EGFR-TKI-resistant NSCLC patients could indeed benefit from ICI plus chemotherapy, but most patients are primary resistant to immunotherapy. Comprehensive analysis of peripheral immune cells using full spectrum flow cytometry showed that compared to the proportion of cell subsets, the expression type and level of ICPs on immune cells, especially CD25, were significantly correlated with the efficacy of immunotherapy. In addition, the prediction model constructed based on features of peripheral immune cells in the present study is promising to screen beneficiaries of EGFR-TKI-resistant NSCLC patients from ICI plus chemotherapy.

## Supplementary Information

**Additional file 1: Supplement table 1.** Detailed information of 23-color-antibody panel designed.

**Additional file 2. Supplement figure 1. **Gating strategy for immune cell subsets.

**Additional file 3: Supplement table 2.** Baseline characteristics of patients whose PBMC samples were collected (PBMC cohort) and comparison of PBMC cohort and the whole study population.

**Additional file 4: Supplement table 3.** Univariate and multivariate analysis of PFS of PBMC cohort.

**Additional file 5. Supplement figure 2. **Heat map of expressions of immune checkpoint proteins in all patients.

## Data Availability

The datasets used and/or analyzed during the current study are available from the corresponding author on reasonable request.

## Abbreviations

APD: Avalanche photodiodes
ARMS-PCR: Amplification refractory mutation system-polymerase chain reaction
AUC: Area under curve
CB: Clinical benefits
CM8: Central memory CD8^+^T cells
CTC: Circulating tumor cells
ctDNA: Circulating tumor DNA
DC: Dendritic cells
DCR: Disease control rate
E4: Effective CD4^+^T cells
ECOG: Eastern Cooperative Oncology Group
EGFR-TKI: Epidermal growth factor receptor-tyrosine kinase inhibitor
EM4: Effective memory CD4 + T cell
HLA-DR: Human leukocyte antigen DR
HR: Hazard ratio
ICI: Immune checkpoint inhibitor
ICPs: Immune checkpoint proteins
LAG-3: Lymphocyte activation gene 3
MDSCs: Myeloid-derived suppressive cells
MFI: Mean fluorescence intensity
NB: Non-benefit
NGS: Next-generation sequencing
NK: Natural killer
NLR: Neutrophil-to-lymphocyte
NOS: Not otherwise specified
NSCLC: Non-small cell lung cancer
ORR: Objective response rate
OS: Overall survival
PD-1: Programmed cell death-1
PD-L1: Programmed cell death ligand 1
PFS: Progression-free survival
PLR: Platelet-to-lymphocyte ratio
PMT: Photomultiplier tube
PR: Partial response
RECIST v1.1: Response Evaluation Criteria in Solid Tumors version 1.1
SD: Stable disease
TC: Tumor cell score
TCR: T cell receptor
TIGIT: T cell immunoglobulin and ITIM domain
TME: Tumor microenvironment
Tregs: Regular T cells
VISTA: V-domain immunoglobulin suppressor of T cell activation
