# Supplementary material for: Full spectrum flow cytometry-powered comprehensive analysis of PBMC as biomarkers for immunotherapy in NSCLC with EGFR-TKI resistance
Source: Biol Proced Online. 2023 Jul 24;25:21. doi: 10.1186/s12575-023-00215-0 (PMC10364374; doi:10.1186/s12575-023-00215-0)
Supplement: Supplementary file 1 — Additional file 1: Supplement table 1. Detailed information of 23-color-antibody panel designed. [file 12575_2023_215_MOESM1_ESM.docx]

| **Supplement table 1. Detailed information of 23-color-antibody panel designed.** | | | | |
| --- | --- | --- | --- | --- |
| **Fluorescein** | **Antibody** | **Manufacturers** | **Code** | **Clone** |
| AF647 | CD183 | Biolegend | 353712 | G025H7 |
| APC | HLA-DR | AAT bioquest | AAT20200330-7 | L243 |
| APC-CY7 | CD3 | Biolegend | 300426 | UCHT1 |
| BB515 | CD4 | BD | 564419 | RPA-T4 |
| SB436 | LAG-3 | Invitrogen | 62-2239-42 | 3DS223H |
| BV480 | CD25 | BD | 566102 | M-A251 |
| BV570 | CD20 | Biolegend | 302332 | 2H7 |
| SB702 | PD-1 | Invitrogen | 67-2799-42 | J105 |
| BV750 | CD56 | Biolegend | 362556 | 5.1H11 |
| FITC | VISTA | Invitrogen | 11-1088-42 | B7H5DSB |
| IF430 | CD16 | AAT bioquest | AAT-20200616-9 | 3G8 |
| IF700 | CD14 | AAT bioquest | AAT20200422-1 | 61D3 |
| MFV540 | CD45RA | AAT bioquest | AAT-20200616-25 | RV526 |
| PE-ef610 | CD197 | Invitrogen | 61-1979-42 | 3D12 |
| PE-cy5 | CD123 | Invitrogen | 15-1239-42 | 6H6 |
| percp-cy5.5 | TIGIT | Biolegend | 372718 | A15153G |
| percp-ef710 | CD127 | Invitrogen | 46-1278-42 | eBioRDR5 |
| PE | CD196 | Invitrogen | 12-1969-42 | R6H1 |
| percp | CD8 | Biolegend | 344708 | SK1 |
| BV605 | CD15 | Biolegend | 323032 | W6D3 |
| percp-if710 | CD11b | AAT bioquest | AAT20200422-2 | ICRF44 |
| SB780 | CD11c | Invitrogen | 78-0016-42 | 3.9 |
| SB645 | CD1c | Invitrogen | 64-0015-42 | L161 |
