# Supplementary figures and images for: Full spectrum flow cytometry-powered comprehensive analysis of PBMC as biomarkers for immunotherapy in NSCLC with EGFR-TKI resistance
Source: Biol Proced Online. 2023 Jul 24;25:21. doi: 10.1186/s12575-023-00215-0 (PMC10364374; doi:10.1186/s12575-023-00215-0)

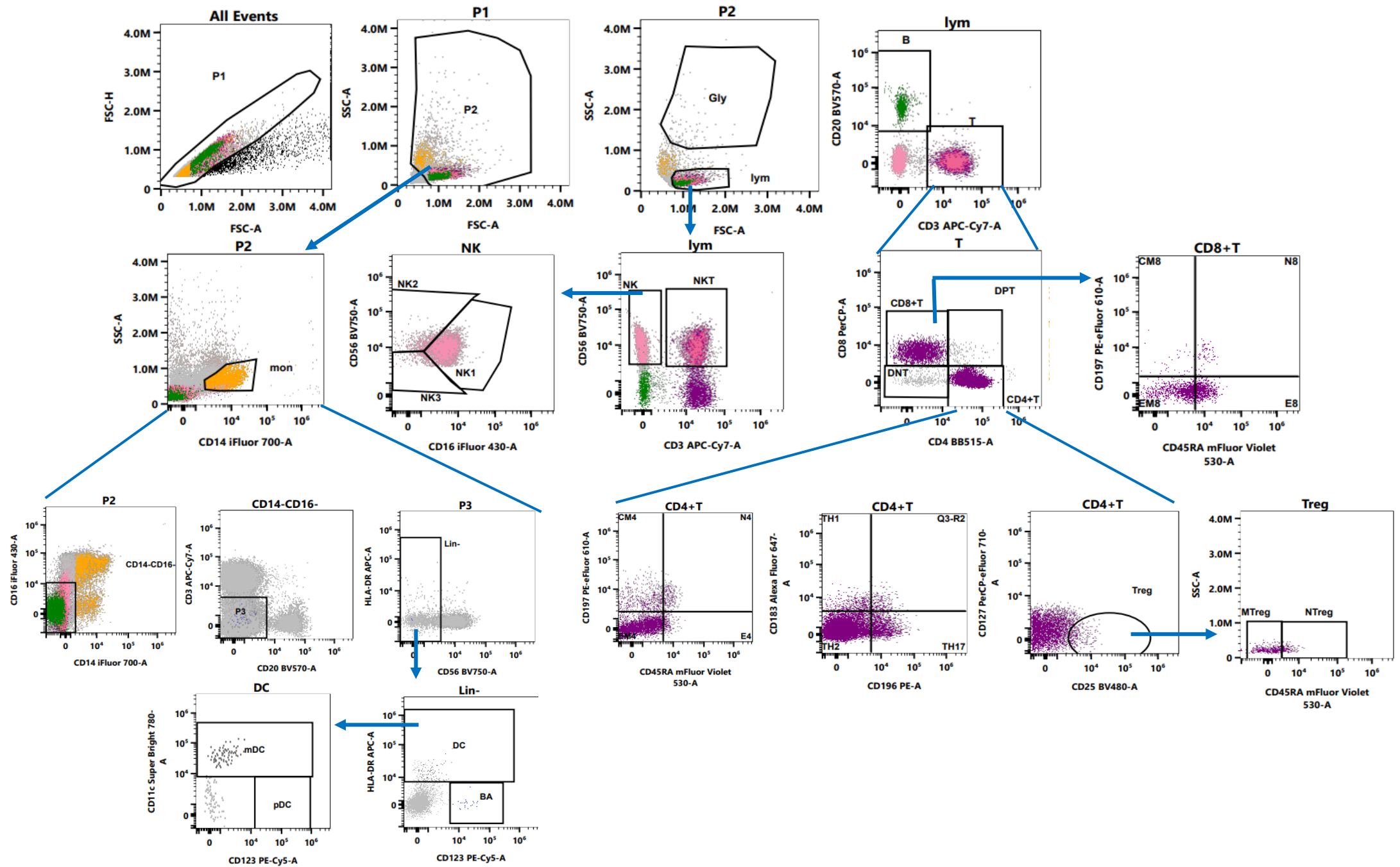

Supplement: Supplementary file 2 — Additional file 2. Supplement figure 1. Gating strategy for immune cell subsets. [file 12575_2023_215_MOESM2_ESM.pdf]
