# Supplementary material for: Full spectrum flow cytometry-powered comprehensive analysis of PBMC as biomarkers for immunotherapy in NSCLC with EGFR-TKI resistance
Source: Biol Proced Online. 2023 Jul 24;25:21. doi: 10.1186/s12575-023-00215-0 (PMC10364374; doi:10.1186/s12575-023-00215-0)
Supplement: Supplementary file 3 — Additional file 3: Supplement table 2. Baseline characteristics of patients whose PBMC samples were collected (PBMC cohort) and comparison of PBMC cohort and the whole study population. [file 12575_2023_215_MOESM3_ESM.docx]

| **Supplement table 2. Baseline characteristics of patients whose PBMC samples were collected (PBMC cohort) and comparison of PBMC cohort and the whole study population** | | | | | | | |
| --- | --- | --- | --- | --- | --- | --- | --- |
| **Characteristics** | **PBMC cohort** | | | |  | **Whole population（N=60）** | ***P*-value** |
|  | **CB (N=9)** | **NB (N=15)** | ***P-value*** | **Total（N=24）** |  |  |  |
| **Age（year）** |  |  | 0.560 |  |  |  | 0.888 |
| median（range） | 59(48-76) | 66(39-76) |  | 63.5(39-76) |  | 63.5(19-76) |  |
| **Sex** |  |  | 1.000 |  |  |  | 0.890 |
| male | 5(55.6) | 7(46.7) |  | 12(50.0) |  | 31(51.7) |  |
| female | 4(44.4) | 8(53.3) |  | 12(50.0) |  | 29(48.3) |  |
| **Histology** |  |  | 0.130 |  |  |  | 0.217 |
| Adeno | 6(66.7) | 14(93.3) |  | 20(83.3) |  | 56(93.3) |  |
| NOS | 3(33.3) | 1(6.7) |  | 4(16.7) |  | 4(6.7) |  |
| **Number of distant metastases** | |  | 1.000 |  |  |  | 1.000 |
| 0-1 | 6(66.7) | 8(53.3) |  | 14(58.3) |  | 35(58.3) |  |
| 2-3 | 3(33.3) | 6(40.0) |  | 9(37.5) |  | 21(35.0) |  |
| ＞3 | 0(0.0) | 1(6.7) |  | 1(4.2) |  | 4(6.7) |  |
| **Organ metastasis** | |  |  |  |  |  |  |
| bone | 2(22.2) | 8(53.3) | 0.210 | 10(41.7) |  | 24(40.0) | 1.000 |
| brain | 3(33.3) | 3(20.0) | 0.635 | 6(25.0) |  | 13(21.7) | 0.741 |
| liver | 1(6.7) | 0(0.0) | 1.000 | 1(4.2) |  | 4(6.7) | 1.000 |
| **EGFR mutation** | |  | 0.598 |  |  |  | 0.609 |
| 19DEL | 4(44.4) | 10(66.7) |  | 14(58.3) |  | 30(50.0) |  |
| L858R | 3(33.3) | 4(26.7) |  | 7(29.2) |  | 27(45.0) |  |
| G719X | 2(22.2) | 1(6.7) |  | 3(12.5) |  | 3(5.0) |  |
| **Acquired T790M** | |  | 0.533 |  |  |  | 0.749 |
| no | 7(77.8) | 14(93.3) |  | 21(87.5) |  | 50(83.3) |  |
| yes | 2(22.2） | 1(6.7) |  | 3(12.5) |  | 10(16.7) |  |
| **Other treatment** | |  | 0.678 |  |  |  | 0.437 |
| no | 5(55.6) | 8(53.3) |  | 13(54.2) |  | 38(63.3) |  |
| yes | 4(44.4) | 7(46.7) |  | 11(45.8) |  | 22(36.7) |  |
| **PD-L1** |  |  | 0.589 |  |  |  | 0.773 |
| not clear | 6(66.7) | 12(80.0) |  | 18(75.0) |  | 52(86.7) |  |
| negative | 2(22.2) | 3(20.0) |  | 5(20.8) |  | 4(6.7) |  |
| positive | 1(11.1) | 0(0.0) |  | 1(4.2) |  | 4(6.7) |  |
| **ICI line** |  |  | 1.000 |  |  |  | 0.195 |
| 2-3 | 6(66.7) | 9(60.0) |  | 16(66.7) |  | 48(80.0) |  |
| >=4 | 3(33.3) | 6(40.0) |  | 8(33.3） |  | 12(20.0) |  |
| **ICI drug** |  |  | 0.527 |  |  |  | 0.766 |
| pembrolizumab | 2(22.2) | 2(13.3) |  | 4(16.7) |  | 12（20.0） |  |
| nivolumab | 0(0.0) | 1(6.7) |  | 1(4.2) |  | 3（5.0） |  |
| camrelizumab | 1(11.1) | 0(0.0) |  | 1(4.2) |  | 7（11.7） |  |
| toripalimab | 0(0.0) | 2(13.3) |  | 2(8.3) |  | 9（15.0） |  |
| tislelizumab | 2(22.2) | 1(6.7) |  | 3(12.5) |  | 6（10.0） |  |
| sintilimab | 1(11.1) | 5(33.3) |  | 6(25.0) |  | 8（13.3） |  |
| not clear | 3(33.3) | 4(26.7) |  | 7(29.2) |  | 15（25.0） |  |
