# Supplementary material for: Full spectrum flow cytometry-powered comprehensive analysis of PBMC as biomarkers for immunotherapy in NSCLC with EGFR-TKI resistance
Source: Biol Proced Online. 2023 Jul 24;25:21. doi: 10.1186/s12575-023-00215-0 (PMC10364374; doi:10.1186/s12575-023-00215-0)
Supplement: Supplementary file 4 — Additional file 4: Supplement table 3. Univariate and multivariate analysis of PFS of PBMC cohort. [file 12575_2023_215_MOESM4_ESM.docx]

| **Supplement table S3.** **Univariate and multivariate analysis of PFS of PBMC cohort.** | | | | | | | |
| --- | --- | --- | --- | --- | --- | --- | --- |
| **Characteristics** | **Univariate** | | |  | **Multivariate** | | |
|  | **HR** | **95%CI** | ***P-value*** |  | **HR** | **95%CI** | ***P-value*** |
| **Age（year）**（<=63.5/>63.5） | 1.155 | 0.475-2.809 | 0.750 |  |  |  |  |
| **Sex** （male/female） | 0.970 | 0.622-1.513 | 0.892 |  |  |  |  |
| **Histology（**adeno/NOS） | 0.659 | 0.213-2.037 | 0.469 |  |  |  |  |
| **Number of distant metastases** | |  |  |  |  |  |  |
| 0-1/>3 | 0.074 | 0.006-0.866 | 0.038 |  |  |  |  |
| 2-3/>3 | 0.123 | 0.011-1.411 | 0.092 |  |  |  |  |
| **Bone metastasis**（no/yes） | 0.447 | 0.176-1.137 | 0.091 |  |  |  |  |
| **Brain metastasis**（no/yes） | 1.096 | 0.388-3.095 | 0.862 |  |  |  |  |
| **Liver metastasis**（no/yes） | 0.094 | 0.009-1.037 | 0.054 |  | 0.094 | 0.009-1.037 | 0.053 |
| **EGFR mutation** |  |  |  |  |  |  |  |
| L858R/19DEL | 0.820 | 0.299-2.247 | 0.700 |  |  |  |  |
| G719X/19DEL | 0.662 | 0.181-2.426 | 0.534 |  |  |  |  |
| **Acquired T790M**（no/yes） | 1.731 | 0.497-6.022 | 0.389 |  |  |  |  |
| **Other treatment**（no/yes） | 0.619 | 0.248-1.544 | 0.304 |  |  |  |  |
| **ICI line**（2-3/>=4） | 0.397 | 0.148-1.067 | 0.067 |  |  |  |  |
| **Lymphocyte**（<=1.07/>1.07） | 1.369 | 0.515-3.639 | 0.528 |  |  |  |  |
| **Neutrophile**（<=3.5/>3.5） | 0.750 | 0.280-2.007 | 0.567 |  |  |  |  |
| **Eosinophils**（<=0.16/>0.16） | 0.724 | 0.237-2.213 | 0.571 |  |  |  |  |
| **Platelet**（<=215/>215） | 0.795 | 0.323-1.955 | 0.618 |  |  |  |  |
| **PLR（<=200/>200）** | 3.417 | 1.067-10.947 | 0.039 |  |  |  |  |
| **NLR（<=4/>4）** | 0.583 | 0.219-1.551 | 0.280 |  |  |  |  |
