# Supplementary material for: Full spectrum flow cytometry-powered comprehensive analysis of PBMC as biomarkers for immunotherapy in NSCLC with EGFR-TKI resistance
Source: Biol Proced Online. 2023 Jul 24;25:21. doi: 10.1186/s12575-023-00215-0 (PMC10364374; doi:10.1186/s12575-023-00215-0)
Supplement: Supplementary file 5 — Additional file 5. Supplement figure 2. Heat map of expressions of immune checkpoint proteins in all patients. [file 12575_2023_215_MOESM5_ESM.pdf]

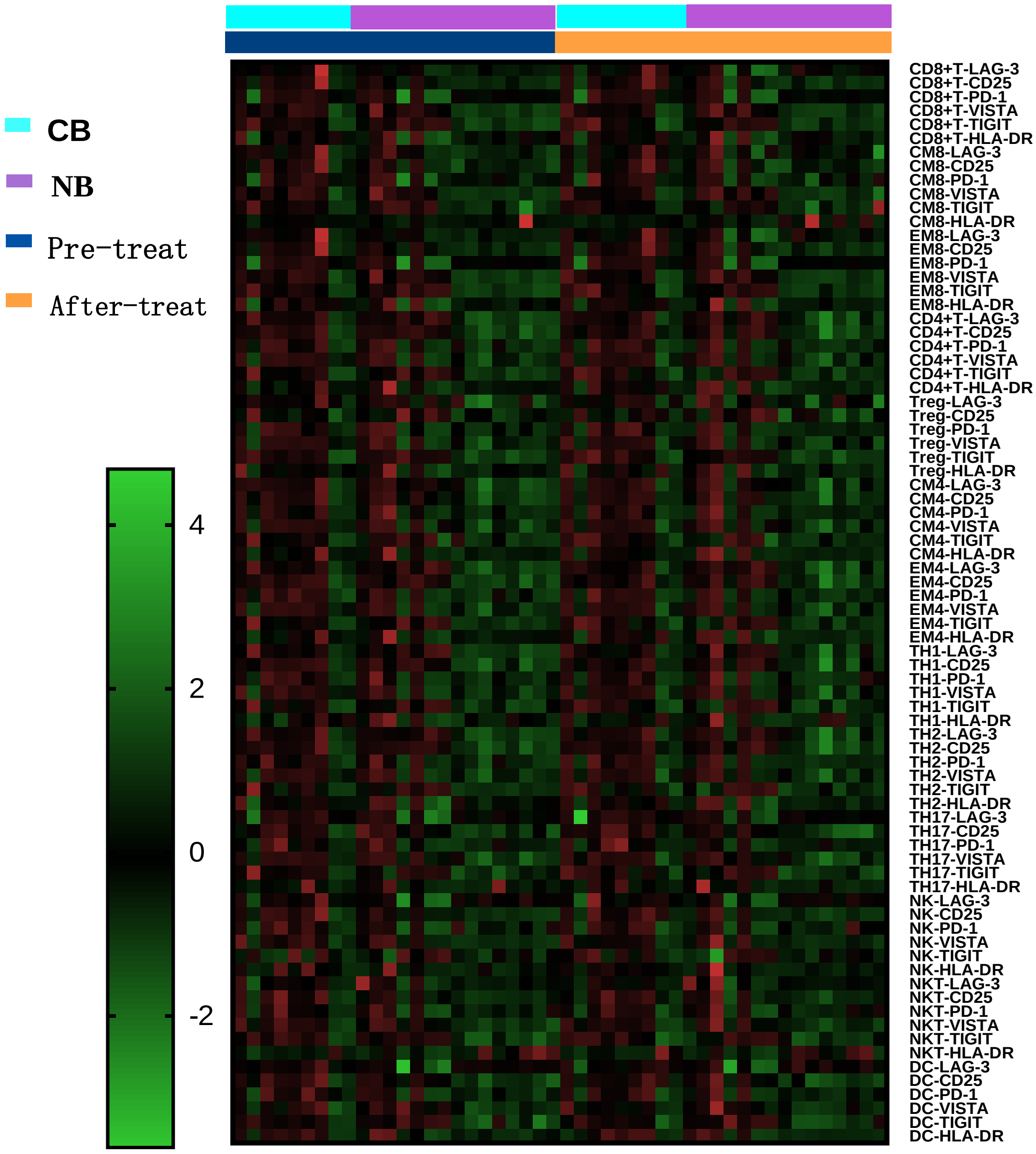
CB
NB
Pre-treat
After-treat
4
2
0
-2
CD8+T-LAG-3
CD8+T-CD25
CD8+T-PD-1
CD8+T-VISTA
CD8+T-TIGIT
CD8+T-HLA-DR
CM8-LAG-3
CM8-CD25
CM8-PD-1
CM8-VISTA
CM8-TIGIT
CM8-HLA-DR
EM8-LAG-3
EM8-CD25
EM8-PD-1
EM8-VISTA
EM8-TIGIT
EM8-HLA-DR
CD4+T-LAG-3
CD4+T-CD25
CD4+T-PD-1
CD4+T-VISTA
CD4+T-TIGIT
CD4+T-HLA-DR
Treg-LAG-3
Treg-CD25
Treg-PD-1
Treg-VISTA
Treg-TIGIT
Treg-HLA-DR
CM4-LAG-3
CM4-CD25
CM4-PD-1
CM4-VISTA
CM4-TIGIT
CM4-HLA-DR
EM4-LAG-3
EM4-CD25
EM4-PD-1
EM4-VISTA
EM4-TIGIT
EM4-HLA-DR
TH1-LAG-3
TH1-CD25
TH1-PD-1
TH1-VISTA
TH1-TIGIT
TH1-HLA-DR
TH2-LAG-3
TH2-CD25
TH2-PD-1
TH2-VISTA
TH2-TIGIT
TH2-HLA-DR
TH17-LAG-3
TH17-CD25
TH17-PD-1
TH17-VISTA
TH17-TIGIT
TH17-HLA-DR
NK-LAG-3
NK-CD25
NK-PD-1
NK-VISTA
NK-TIGIT
NK-HLA-DR
NKT-LAG-3
NKT-CD25
NKT-PD-1
NKT-VISTA
NKT-TIGIT
NKT-HLA-DR
DC-LAG-3
DC-CD25
DC-PD-1
DC-VISTA
DC-TIGIT
DC-HLA-DR
